# Comparative Study of Intravenous Dexmedetomidine Sedation With Perineural Dexmedetomidine on Supraclavicular Approach Brachial Plexus Block in Upper Limb Orthopaedic Surgery

**DOI:** 10.7759/cureus.10768

**Published:** 2020-10-02

**Authors:** Priyank Samar, Tanvi A Dhawale, Sarla Pandya

**Affiliations:** 1 Anesthesiology, K.J. Somaiya Medical College and Hospital, Mumbai, IND

**Keywords:** supraclavicular block, bupivacaine, lignocaine, dexmedetomidine, upper limb surgery

## Abstract

Background

Dexmedetomidine is being used as an adjuvant analgesic, both as intravenous (IV) and intrathecal infusion. The role of perineural (P) dexmedetomidine has evoked attention recently. The aim of this study was to compare the effect of IV dexmedetomidine and P dexmedetomidine as an adjunct to supraclavicular brachial plexus block in upper limb orthopaedic surgery.

Methods

Patients were randomly divided into two equal groups (n=20). Group I (IV dexmedetomidine) received dexmedetomidine 1 mcg/kg IV as loading dose over 10 minutes, followed by continuous infusion of dexmedetomidine 0.4 mcg/kg/hr IV. Group P (P dexmedetomidine) received dexmedetomidine at 1 mcg/kg perineurally. After adequate motor response with the aid of peripheral nerve stimulator a supraclavicular block with 40 ml solution containing 5 mg/kg lignocaine (2%) with adrenaline (1:200,000) and 2 mg/kg of bupivacaine (0.5%) was injected to both the groups. Group P also received dexmedetomidine perineurally with block. Onset and duration of sensory and motor block, Ramsay sedation score, hemodynamic parameters, and postoperative analgesia requirement were assessed along with side effects. The data obtained were recorded as mean ± SD, ranges, numbers, and ratios. Results were analyzed using the chi-square test, the Mann-Whitney test for non-parametric data, and an unpaired ‘t’-test for parametric data. Statistical analysis was carried out using the SPSS (version 10, 2002; SPSS Inc., Chicago, IL, USA) for Windows statistical package. P value less than 0.05 was considered statistically significant.

Results

Mean onset of sensory block was earlier in group I than in group P (p<0.05) although mean onset of motor block was not significantly different (p>0.05). Duration of sensory and motor blockade was longer in group I (p<0.05). Patients in group I demonstrated lower pulse rate and lower systolic and diastolic blood pressures throughout the period with comparable SpO_2_ values. There was no difference in intraoperative Ramsay sedation scores in both groups, but postoperative Ramsay sedation scores at 9, 12, and 15 hours were better in group I (p<0.05). The average time to rescue analgesia (visual analogue scale >4) was higher in group I (p>0.05).

Conclusion

IV dexmedetomidine produced early onset of sensory block, longer duration of sensory and motor block, and longer duration of analgesia as compared with P dexmedetomidine as an adjuvant to supraclavicular block with 5 mg/kg lignocaine (2%) and 2 mg/kg bupivacaine (0.5%) in upper limb orthopaedic surgeries.

## Introduction

Orthopaedic surgical procedures are often painful, and analgesic alternatives to general anaesthesia and intravenous (IV) opioid pain therapy are important. Brachial plexus blockade is a method of regional anaesthesia used in hand, forearm, and arm surgeries using various anatomical approaches, such as interscalene, supraclavicular, infraclavicular, and axillary approaches [1-3]. Systemic administration of alpha 2 agonists has been reported to induce sedative effects and reduce opioid requirements in the perioperative period providing evidence that these drugs have an analgesic action that may be mediated by both supraspinal mechanisms in the locus coeruleus and spinal mechanisms in the dorsal horn of the spinal cord [4,5]. Dexmedetomidine is a highly selective alpha 2 adrenergic receptor agonist most often used for short-term sedation in patients on mechanical ventilation in intensive care because it does not induce major respiratory side effects [6]. It has been found to have an antihyperalgesic action in rats with neuropathic pain originating in the peripheral nervous system and enhances the effects of analgesics without increasing side effects. The analgesic, sedative/hypnotic, and anxiolytic properties and its opioid-sparing effect make dexmedetomidine potentially useful for painful surgical procedures [7,8].

This comparative prospective randomized study aimed to assess the effect of IV and perineural dexmedetomidine as adjuvant to supraclavicular brachial plexus block in upper limb orthopaedic surgery.

## Materials and methods

After obtaining approval from Institutional Ethics Committee, this comparative prospective randomized study was planned with 40 patients ranging in age from 18 to 60 years of American Society of Anaesthesiologists (ASA) I and II, weighing 40-70 kg who were undergoing upper extremity orthopaedic surgeries. Patients with a history of bleeding diathesis, cervical rib, shoulder joint pathology, or allergy to the drug used, history or presence of cardiac, respiratory, and/or renal failures, and those who were pregnant were not included in the study. Patients were divided into two equal groups (n=20): group I was assigned to receive IV dexmedetomidine 1 mcg/kg as loading dose over 10 minutes from 50 ml infusion syringe, this was followed by continuous infusion of dexmedetomidine starting at 0.4 mcg/kg/hr IV; group P was assigned to receive perineural dexmedetomidine at 1 mcg/kg. Sealed envelope technique was used for assignment. An IV cannula was inserted into the contralateral arm and a 5 ml/kg/hr infusion of Ringer acetate solution was started. All patients were monitored non-invasively with an ECG, non-invasive blood pressure measurement, and pulse oximetry (SpO_2_). Patients were positioned supine with the arm abducted to create 90 degrees angle with the body, and the forearm was flexed and externally rotated so that the hand could be placed next to the head and the palm could be positioned facedown. Then, the supraclavicular region was disinfected and a 22 G 5-cm Stimuplex® needle (B. Braun Medical Inc, Bethlehem, PA) was inserted into the supraclavicular region under sterile conditions. 

Nerves were identified through stimulation of muscle supplied by a certain nerve to contract by application of a current of 1 mA, which is adequate to cause contraction without being painful, using a nerve stimulator through the Stimuplex needle. Nerve identification was carried out in the order radial (extension and supination of the arm and fingers), median (flexion and pronation of the wrist, second, and third fingers), ulnar (flexion of the fourth and fifth fingers and thumb adduction), and musculocutaneous nerves (arm flexion). Once the desired response was found, the needle was stabilized, and 40 ml of solution of local anaesthetic containing 5 mg/kg lignocaine (2%) with adrenaline (1:200,000) and 2 mg/kg of bupivacaine (0.5%) was injected to both groups. Group P also received the perineural dexmedetomidine with block.

Onset of sensory block was the time from injection to the onset of analgesia in each of the major peripheral nerve distribution (ulnar, radial, medial, musculocutaneous). Duration of sensory blockade was the time elapsed between injection of the drug and appearance of visual analogue score (VAS)>3. Onset of motor block was the time from injection to the complete loss of flexion at elbow (musculocutaneous nerve), extension of the elbow and wrist (radial nerve), opposition of thumb and index finger (median nerve), and opposition of thumb and little finger (ulnar nerve). Duration of motor blockade was the time elapsed between injection of the drug to complete return of motor power. The following parameters were recorded: onset and duration of sensory and motor block, haemodynamic parameters (pulse rate/blood pressure/SpO_2_), Ramsay sedation score (1-4), postoperative analgesia requirement, and side effects. The duration of analgesia was defined as the time elapsed from the end of the block till the first request for analgesia. The severity of pain at the time of request of rescue analgesia was rated using a 10-point VAS, with 0 indicating no pain and 10 indicating worst imaginable pain. Rescue analgesia was provided in the form of diclofenac 75 mg intravenously when VAS was >4. 

Statistical analysis 

Power analysis was done for sample size with confidence limits at 95% and power at 80% to detect a minimum 10% difference in degree of sensory/motor blockade between groups. The data obtained were presented as mean ± SD, ranges, numbers, and ratios. Results were analyzed using the chi-square test, the Mann-Whitney test for non-parametric data, and an unpaired ‘t’-test for parametric data. Statistical analysis was carried out using the SPSS (version 10, 2002; SPSS Inc., Chicago, IL, USA) for Windows statistical package. P value less than 0.05 was considered statistically significant. 

## Results

The study included 40 patients, 18-60 years, ASA status 1 and 2, weight between 40 and 70 kg. The groups were similar in terms of age, sex, and duration of surgery (Table 1).

**Table 1:** Comparison of group I and group P *p<0.05 is significant ASA, American Society of Anaesthesiologists; VAS, visual analogue scale

	Group I	Group P	P value
Mean age, years	31.5±12	32.03±10	>0.05
Mean weight	52±10	51±10	>0.05
Sex, male: female	15:5	16:4	>0.05
ASA I:II	11:9	13:7	>0.05
Mean onset sensory block in minutes	2.6±1.2	3.2±1.4	<0.05*
Mean onset motor block in minutes	6.3±2.5	6.7±3.1	>0.05
Mean duration of sensory block in minutes	670±100	540±94	<0.05*
Mean duration of motor block in minutes	800±110	600±105	<0.05*
Average time to rescue analgesia in minutes, VAS >4	1320±276	1158±264	>0.05

Mean onset of sensory block was earlier in group I than in group P (p<0.05) although mean onset of motor block was not significantly different (p>0.05).

Duration of sensory and motor block was significantly longer in group I than in group P (p<0.05) (Table 1).

**Figure 1:**
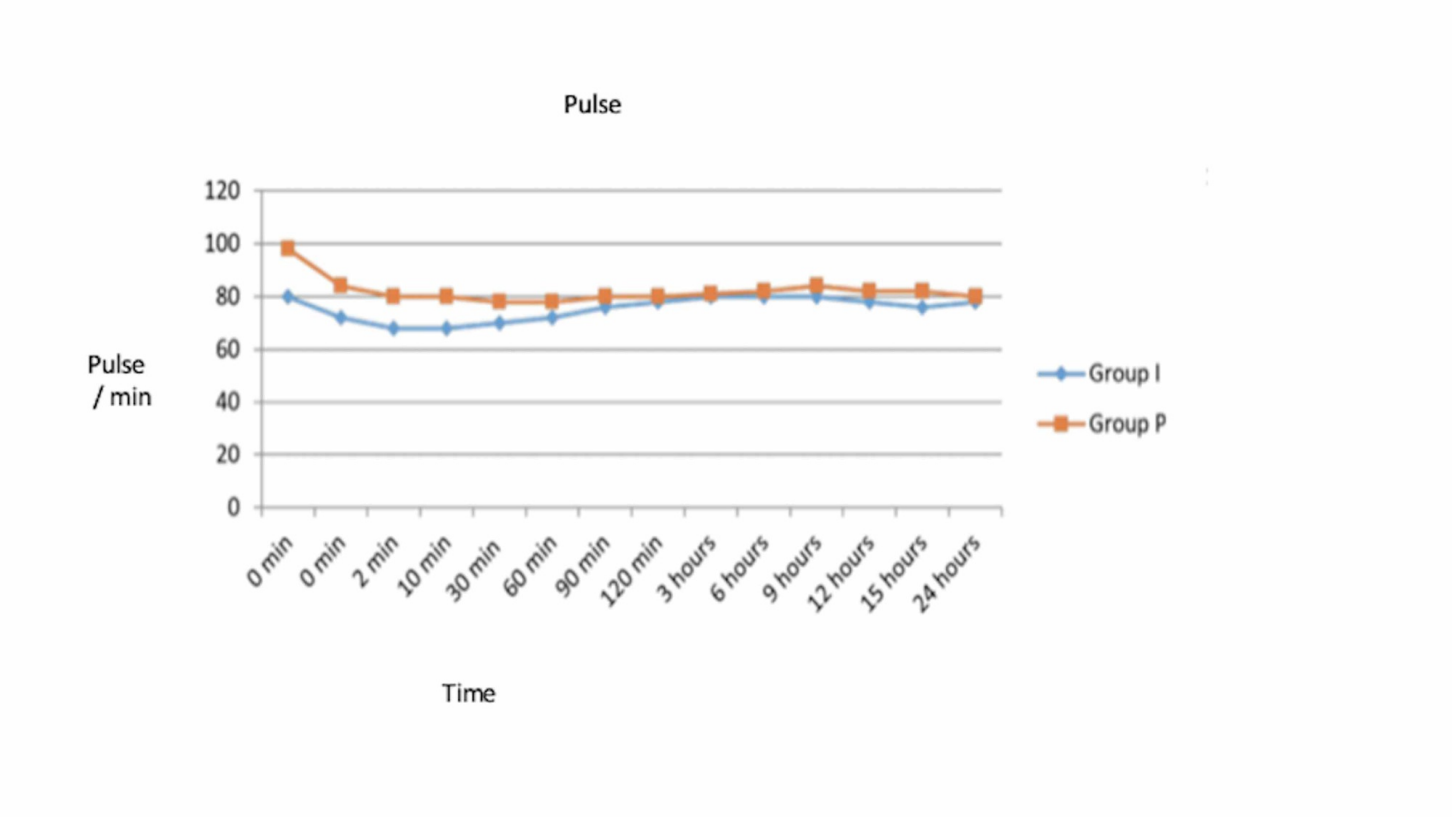
Mean pulse at various time intervals in group I and group P

**Figure 2:**
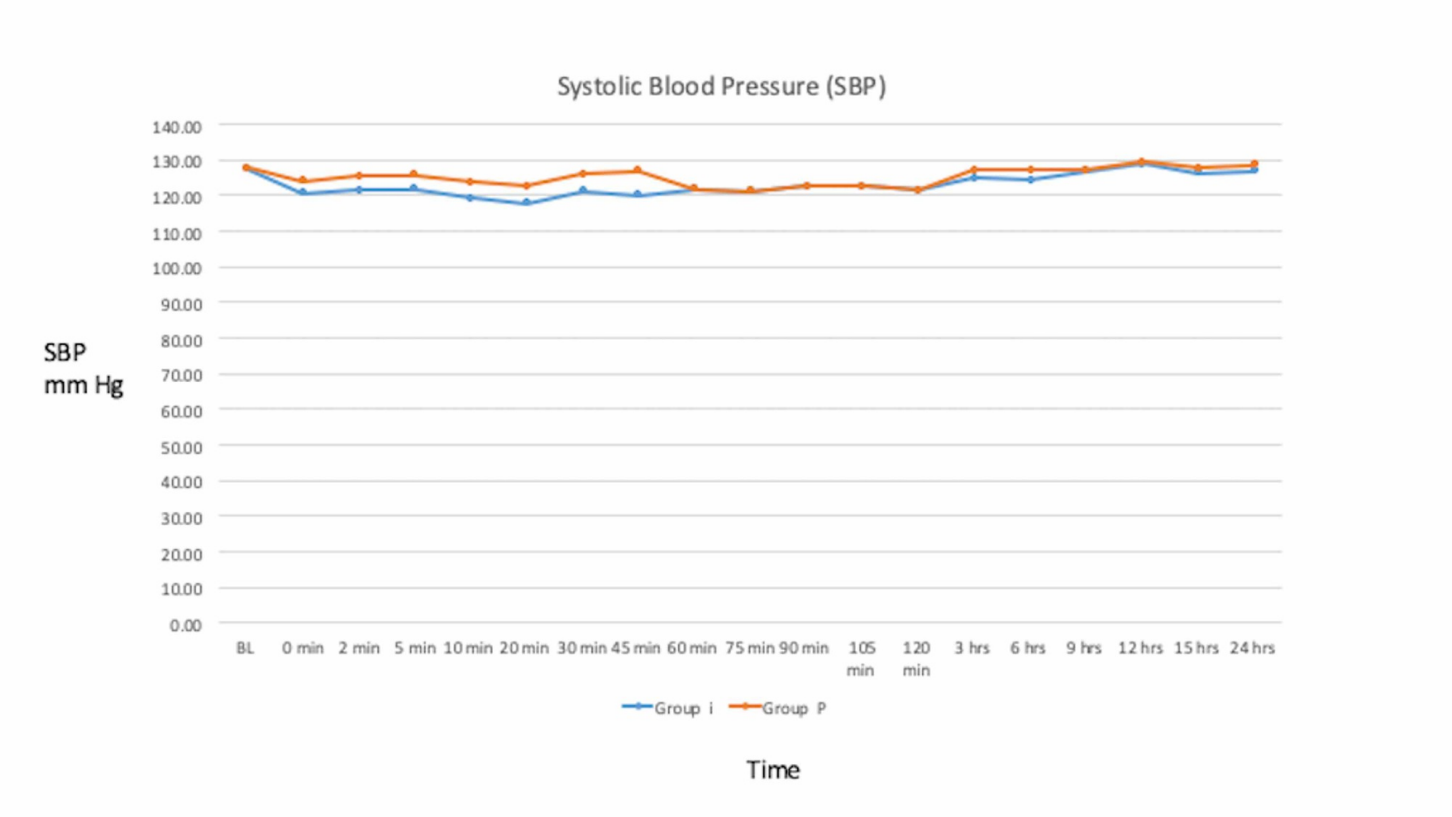
Mean systolic blood pressure (SBP) in group I and group P at various time intervals

**Figure 3:**
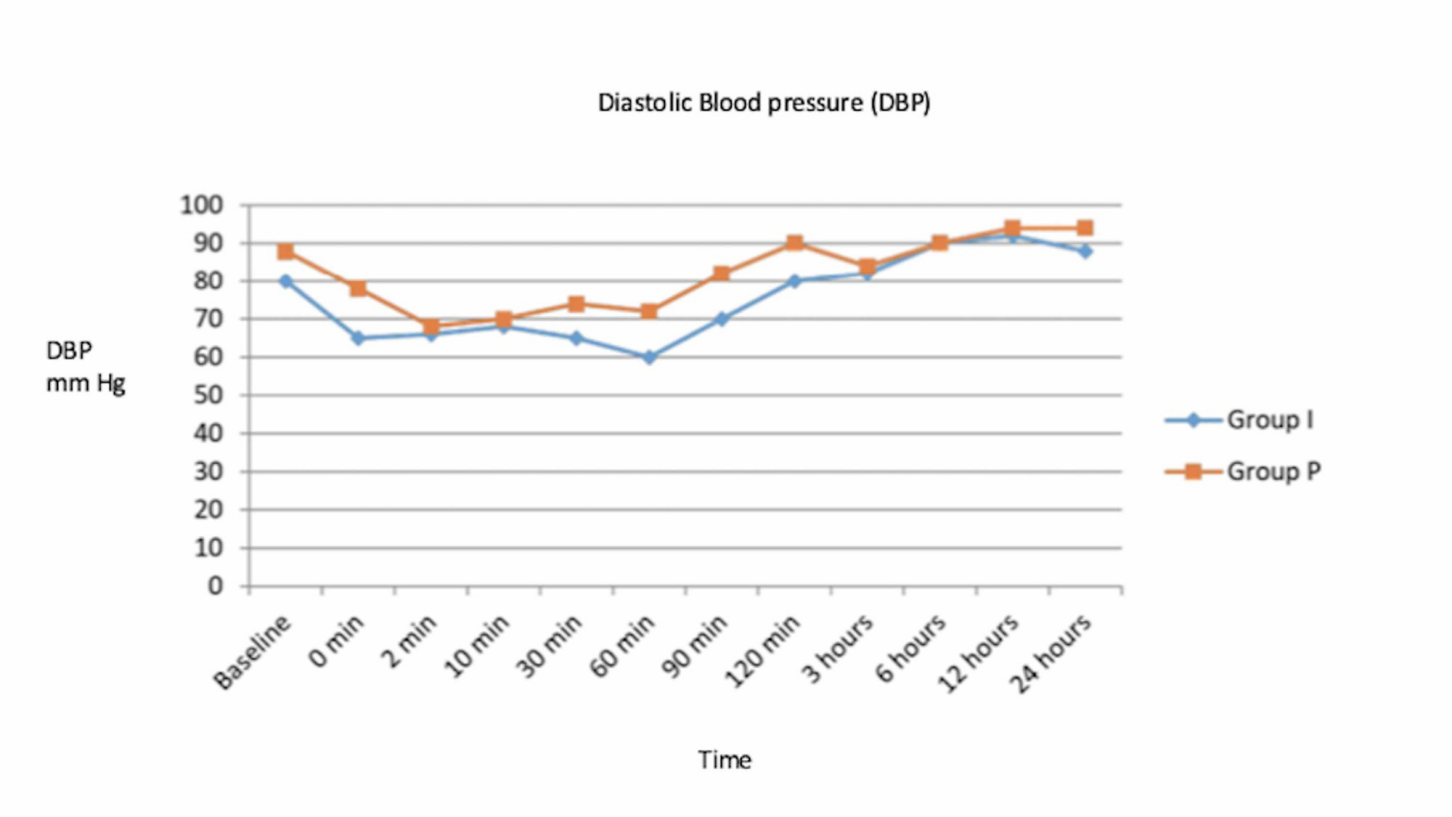
Mean diastolic blood pressure (DBP) changes in group I and group P at various time intervals

Patients in group I demonstrated lower pulse rate and lower systolic and diastolic blood pressures as compared to group P throughout the period with comparable SpO_2_ values (Figures 1-3).

 

Side effects 

Three patients from group I had bradycardia that was asymptomatic and responded to IV atropine 0.6 mg. There was no significant difference in intraoperative Ramsay sedation scores in both the groups (p>0.05). Postoperative Ramsay sedation scores at 9, 12, and 15 hours were significantly better in group I than in group P (p<0.05). The average time to rescue analgesia (VAS>4) was not significantly different between the groups (p>0.05) (Table 1).

There was no significant difference between both groups in terms of onset of motor block, average time to rescue analgesia, and in intraoperative Ramsay sedation scores. However, the sensory onset time was significantly earlier and the duration of sensory and motor block was significantly longer in group I compared with group P. 

## Discussion

The results obtained indicated a satisfactory outcome of supraclavicular block for upper limb surgeries that allowed for management as an outpatient procedure and minimization of postoperative analgesia.

Few studies have evaluated the analgesic effects of dexmedetomidine on regional administration [9-13]. Al-Mustafa et al. also found that dexmedetomidine exerts a dose-dependent effect on the onset and regression of sensory and motor block when used as an adjuvant to bupivacaine in spinal anaesthesia [9]. El-Hennawy et al. found that the addition of dexmedetomidine or clonidine to caudal bupivacaine significantly prolonged the analgesia time than use of bupivacaine alone [10]. Mizrak et al. compared the effects of dexmedetomidine 0.5 mcg/kg when added to lidocaine for IV regional anaesthesia and reported significantly reduced sensory and motor block onset times, recovery time, and decreased intra and postoperative VAS scores and analgesic requirement, and concluded that addition of dexmedetomidine to lidocaine and premedication for IV regional anaesthesia similarly improved the quality of anaesthesia and perioperative analgesia without major side effects [11]. Obayah et al. evaluated the effect of adding dexmedetomidine to bupivacaine on the duration of postoperative analgesia in children who underwent repair of a cleft palate using a combination of general anaesthesia and greater palatine nerve block with a combination of dexmedetomidine and bupivacaine, and reported increased duration of analgesia after repair of a cleft palate by 50%, with no clinically relevant side effects [12]. Esmaoglu et al. found that dexmedetomidine added to levobupivacaine for an axillary brachial plexus block reduces the onset time and prolongs the duration of the block and the duration of postoperative analgesia [13]. The results obtained were comparable and superior to those obtained with other local anaesthetic adjuvant. Movafegh et al. found that the addition of an ultralow dose of naloxone to a lidocaine 1.5% solution with or without Fentanyl solution in an axillary brachial plexus block prolongs the time to first postoperative pain and motor blockade but also increases the onset time [14]. Sarsu et al. found that addition of 100 mg of tramadol to the combination of levobupivacaine and lidocaine during an axillary brachial block did not induce a major clinical effect in patients undergoing hand and forearm surgery [15]. Samy et al. found that combination of bupivacaine and dexmedetomidine in axillary block provided satisfactory perioperative anaesthesia and analgesia and allowed the management of upper limb orthopaedic surgery as an outpatient procedure [16]. 

The limitations are the relatively small series and different upper limb orthopaedic procedures and lack of blinding. 

## Conclusions

IV dexmedetomidine produced early onset of sensory block, longer duration of sensory and motor block, and longer duration of analgesia as compared with perineural dexmedetomidine as an adjuvant to supraclavicular block with 5 mg/kg lignocaine (2%) and 2 mg/kg bupivacaine (0.5%) in upper limb orthopaedic surgeries. There were no significant differences in terms of onset of motor block, average time to rescue analgesia, and in intraoperative Ramsay sedation scores with IV or perineural dexmedetomidine. Three patients in the IV dexmedetomidine group had asymptomatic bradycardia.

A larger prospective study with a single anatomical approach for the block and surgical procedure is recommended for further evaluation of this subject.

## References

[1] De Tran QH, Clemente A, Doan J, Finlayson RJ (2007). Brachial plexus blocks: a review of approaches and techniques. Can J Anaesth.

[2] Jafari S, Kalstein AI, Nasrullah HM, Hedayatnia M, Yarmush JM, SchianodiCola J (2008). A randomized, prospective, double-blind trial comparing 3% chloroprocaine followed by 0.5% bupivacaine to 2% lidocaine followed by 0.5% bupivacaine for interscalene brachial plexus block. Anesth Analg.

[3] Klaastad O, Sauter AR, Dodgson MS (2009). Brachial plexus block with or without ultrasound guidance. Curr Opin Anaesthesiol.

[4] Marinangeli F, Ciccozzi A, Donatelli F (2002). Clonidine for treatment of postoperative pain: a dose finding study. Eur J Pain.

[5] Arain SR, Ruehlow RM, Uhrich TD, Ebert TJ (2004). The efficacy of dexmedetomidine versus morphine for postoperative analgesia after major inpatient surgery. Anesth Analg.

[6] Cortinez LI, Hsu YW, Sum-Ping ST (2004). Dexmedetomidine pharmacodynamics: part II. Crossover comparison of the analgesic effect of dexmedetomidine and remifentanil in healthy volunteers. Anesthesiology.

[7] Unlugenc H, Gunduz M, Guler T, Yagmur O, Isik G (2005). The effect of pre-anesthetic administration of intravenous dexmedetomidine on postoperative pain in patients receiving patient-controlled morphine. Eur J Anaesth.

[8] Bulow NM, Barbosa NV, Rocha JB (2007). Opioid consumption in total intravenous anesthesia is reduced with dexmedetomidine: a comparative study with remifentanil in gynecologic videolaparoscopic surgery. J Clin Anesth.

[9] Al-Mustafa MM, Abu-Halaweh SA, Aloweidi AS (2009). Effect of dexmedetomidine added to spinal bupivacaine for urological procedures. Saudi Med J.

[10] El-Hennawy AM, Abd-Elwahab AM, Abd-Elmaksoud AM, El-Ozairy HS, Boulis SR (2009). Addition of clonidine or dexmedetomidine to bupivacaine prolongs caudal analgesia in children. Br J Anaesth.

[11] Mizrak A, Gul R, Erkutlu I, Alptekin M, Oner U (2010). Premedication with dexmedetomidine alone or together with 0.5% lidocaine for IVRA. J Surg Res.

[12] Obayah GM, Refaie A, Aboushanab O, Ibraheem N, Abdelazees M (2010). Addition of dexmedetomidine to bupivacaine for greater palatine nerve block prolongs postoperative analgesia after cleft palate repair. Eur J Anaesthesiol.

[13] Esmaoglu A, Yegenoglu F, Akin A, Turk CY (2010). Dexmedetomidine added to levobupivacaine prolongs axillary brachial plexus block. Anesth Analg.

[14] Movafegh A, Nouralishahi B, Sadeghi M, Nabavian O (2009). An ultra-low dose of naloxone added to lidocaine or lidocaine-fentanyl mixture prolongs axillary brachial plexus blockade. Anesth Analg.

[15] Sarsu S, Mizrak A, Karakurum G (2011). Tramadol use for axillary brachial plexus blockade. J Surg Res.

[16] Hanoura SE, Elsayed MM, Abdullah AA, Elsayed HO, Nor Eldeen TM (2013). Dexmedetomidine improves the outcome of a bupivacaine brachial plexus axillary block: a prospective comparative study. Ain-Shams J Anesthesiol.

